# A Rapid and Reliable Propidium Monoazide Polymerase Chain Reaction for Detecting Viable *Pseudomonas syringae* pv. *actinidiae*

**DOI:** 10.3390/cimb47020103

**Published:** 2025-02-06

**Authors:** Yi Luo, Wenfei Liao, Yue Li, Wen Chen, Sen Zhong, Cuiping Wu, Kaikai Yao, Rui Yang, Miaomiao Ma, Guoshu Gong

**Affiliations:** Plant Protection Department and Major Crop Disease Laboratory, College of Agronomy, Sichuan Agricultural University, Chengdu 611130, China; luoyixinlang@sina.cn (Y.L.); 2021201059@stu.sicau.edu.cn (W.L.); 2021301140@stu.sicau.edu.cn (Y.L.); 2021301154@stu.sicau.edu.cn (W.C.); zhongsen@lomonbio.com (S.Z.); 71262@sicau.edu.cn (C.W.); yaokaikai@stu.sicau.edu.cn (K.Y.); 2020201073@stu.sicau.edu.cn (R.Y.); mmma@sicau.edu.cn (M.M.)

**Keywords:** kiwifruit canker disease, *Pseudomonas syringae* pv. *actinidiae*, propylazide bromide, PCR, viable bacteria

## Abstract

*Pseudomonas syringae* pv. *actinidiae* (Psa) is responsible for causing kiwifruit canker disease. The detection of Psa is commonly carried out using normal PCR and culture-based isolation. However, normal PCR does not differentiate between live and dead cells, potentially resulting in the incorrect estimation of the amount of infectious substance in a sample. Such an incorrect estimation could result in unnecessary phytosanitary strategies and control measures. This study attempts to establish a specific assay for detecting only live Psa bacterial cells. To achieve this, a pair of strain-specific primers designed from *HopZ3* effector were used, and the traditional PCR method was assessed using a nucleic acid-binding dye (propidium monoazide—PMA), establishing a PMA–PCR system and conditions for detecting live Psa in this study. Sensitivity tests showed a detection limit of 10 cfu/mL and 1 pg/μL. This method was also tested in diseased kiwifruit tissues and can be seen as a rapid and dependable replacement to PCR methods for detecting only those infective kiwifruit materials with viable Psa.

## 1. Introduction

Kiwifruit (*Actinidia chinensis* Planch) is one of the most successful artificial domestication and cultivation fruit crops of the 21st century. In 1904, kiwifruit was introduced to New Zealand from China and was subsequently widely cultivated across New Zealand [1]. After more than 100 years, kiwifruit has evolved from a domesticated wild plant to a commercial crop with international economic importance [1]. The cultivation acreage and annual production for kiwifruit have increased gradually in recent years, and kiwifruit bacterial canker disease is one of the most important diseases affecting the kiwifruit planting industry. It mainly causes dried and cracked branches (e.g., trunk, cane, and vine), withered and falling leaves, brown flowers that are typically dried without pollination, and fruit-pitted rot, seriously affecting the quality and yield of the fruit [2]. Consequently, these symptoms lead to a serious economic loss for the kiwifruit industry globally [3,4]. For instance, it was reported that, by 2014, this disease had led to direct economic losses reaching up to NZD 560 million in New Zealand [4]. The causal agent of bacterial canker disease in kiwifruit is *Pseudomonas syringae* pv. *actinidiae*, which is a national forest quarantine pest [5]. Psa populations are currently divided into five pathogenic biovars (namely, Psa1, Psa2, Psa3, Psa5, and Psa6) based on genetic diversity, pathogenicity, and toxin production [6,7,8,9]. Biovar 4, which is only distributed in partial areas of New Zealand and Australia, was renamed as *Pseudomonas syringae* pv. *actinidifoliorum* (Pfm) in 2015 [10]. Since 2010, Psa3 has been a pandemic group and can cause severe canker disease around the world [3,11,12,13,14].

Because of the huge influence caused by kiwifruit canker disease, there is an urgent need to establish an effective control strategy. In general, chemical control has little effect on kiwifruit canker disease once the symptoms have appeared [15,16]. Likewise, it is impossible to effectively control the disease in the late infection period [15]. The most important task in preventing this disease is to detect its pathogenic bacteria. Therefore, the timely detection of the pathogen is key to control the disease successfully, in order to guarantee the sanitation and health situation to protect orchards as early as possible. Many detection techniques have been applied to detect the pathogen, such as specific-primer PCR, duplex PCR, multiplex PCR, nested PCR, real-time quantitative PCR, droplet digital PCR, serological immunoassays, and recombinase polymerase amplification-lateral flow (RPA-LF) [16,17,18,19,20,21,22,23,24]. However, there are few reports about the detection of viable bacteria. Moreover, the traditional PCR detection methods are prone to false positive results [25], leading to many unnecessary losses due to the wrong prevention and control strategies. However, direct plate culture is time-consuming and laborious. Although the RT-PCR method based on mRNA in prokaryotes can effectively distinguish false positive samples, the results are prone to be affected by multiple factors because of the relatively complicated operating steps and the instability of mRNA, resulting in great differences in the detection of actual samples [26].

A photosensitive DNA dye, propidium monoazide (PMA), is unable to penetrate into the cell membrane of a complete cell [27]. However, it can effectively modify the DNA exposed after cell death, forming stable covalent carbon–nitrogen bonds and preventing the amplification of these DNA molecules via PCR. In addition, the remaining PMA after photolysis reacts with water molecules to produce inactive hydroxylamine, which enables the DNA in dead cells to be distinguished from that in living cells, effectively detecting the live bacteria in the sample [27]. The target of PMA–PCR detection is live bacterial DNA molecules, which avoids the problem of false positives in traditional PCR detection and makes the results more reliable and effective. The PMA–PCR assay has been widely utilized in the detection of various plant pathogens [28,29,30,31,32,33,34,35]. For example, Panth et al. [36] developed an assay using PMA to specifically detect live *Xanthomonas arboricola* pv. *pruni* in peaches, providing a good tool for detecting overwintering pathogenic bacteria in peach trees. Wang et al. [37] and Immanuel et al. [38] have also successfully detected the pathogen *Xanthomonas fragariae* in strawberry using PMA. Based on this, our study combined PMA and PCR technology to develop a PMA–PCR method for accurately and rapidly detecting live Psa cells and analyzed the influence of PMA on the traditional PCR detection of Psa so as to monitor the early infection and real dynamic changes in the Psa population as well as provide technical support for inspection, quarantine, and disease control.

## 2. Materials and Methods

### 2.1. Bacterial Strains and Culture

Ten identified Psa strains (MJ2107, CX1101, AB2101, DH2101, DS2102, DF2101, MP2101, MN2101, QL0102, and YA1801) (Table A1) and three *Pseudomonas* spp. (*P. fluorescens*, *P*. *viridiflava*, and *P. lurida*) were selected for the specific detection of primers, which were isolated from kiwifruit tissues in the Sichuan province of China. Seven different pathogenic variants of *Pseudomonas syringae* populations (*P*. *syringae* pv. *tomato* DC3000, *P*. *syringae* pv. *syringae*, *P*. *syringae* pv. *theae*, *P*. *syringae* pv. *lachrymans*, *P*. *syringae* pv. *tabaci*, *P*. *syringae* pv. *mori*, *and P*. *syringae* pv. *morsprunorum*) were also included. All tested bacterial strains were stored with 25% glycerol at −80 °C and were then cultured on Luria–Bertani (LB) agar medium at 25 °C for 48 h before further progress.

Among these strains, Psa strain MJ2107 (deposited in the laboratory of Plant Protection Department, College of Agriculture, Sichuan Agricultural University), isolated from the leaves of infected kiwifruit showing obvious symptoms in Mianzhu city of Sichuan Province, was used in all the assays. It was grown at 25 °C with a 250 rpm/min shaker for 14 h in LB broth and was then plated using streak culture on LB agar medium. A single colony was selected and inoculated into a 50 mL centrifuge tube containing 20 mL LB broth, cultured overnight at 180 rpm/min in a shaker at 25 °C until the OD_600_ reached 1.5~2.0, and then re-suspended with sterile water and adjusted to a final concentration of 10^7^ colony forming unit (cfu)/mL suspensions using a spectrophotometer for further study. The lethal temperature was set at 100 °C and was heated for 11 to 20 min at 1 min increments. After being cooled on ice, an additional 100 µL of the treated suspensions was subjected to spread plate cultivation on LB agar medium for two weeks to determine whether the bacterial cells were killed and dead.

### 2.2. DNA Extraction of the Tested Bacterial Strains

A single colony of all the tested bacteria was grown overnight in LB broth at 25 °C in a 200 rpm/min shaker until the OD_600_ reached 2.0~2.5, and the bacterial precipitate was collected in a Sorvall Legend Micro 17 benchtop centrifuge (Thermo Fisher Scientific, Waltham, MA, USA) at 13,000× *g* for 1 min. The genomic DNA of each treated bacterium was extracted using the TIANamp Bacteria DNA Kit (TIANGEN Biotech Co., Ltd., Beijing, China), according to its usage instructions. DNA concentration and quality were quantified using a Nanodrop 2000 spectrophotometer (Thermo Fisher Scientific, Waltham, MA, USA) before being adjusted to 50 ng/mL with sterile distilled water and stored at −20 °C.

### 2.3. Comparison of Primer Amplification Sensitivity and Specificity

The genomic DNA concentration of Psa strain MJ2107 was gradient-diluted to 10 ng/μL, 1 ng/μL, 100 pg/μL, 10 pg/μL, 1 pg/μL, 100 fg/μL, 10 fg/μL, and 1 fg/μL successively. The bacterial suspension with a concentration of 10^7^ cfu/mL was gradient-diluted 10-fold until it reached 10^0^ cfu/mL. Two pairs of primers, PsaF/R (designed for *HopZ3* effector) [18] and HopZ5F/R (designed for *HopZ5* effector) [39], were used for detecting only Psa in this study (Table 1). PCR was carried out in a 25 μL total volume mixture containing 2.5 μL of 10×Taq buffer, 1 μL of 25 mM MgCl_2_, 1 μL of Taq DNA Polymerase (Vazyme Biotech, Nanjing, China), 1 μL of 2.5 mM dNTP mix, 1 μL of template DNA, 1 μL of 10 μM primers PsaF/R or HopZ5F/R, and sterile distilled water to a final volume. Amplification conditions were performed in an S1000 thermal cycler (Bio-Rad Laboratories, Shanghai, China). The amplification protocol for the HopZ5F/R primer was performed as follows [39]: an initial denaturation step at 95 °C for 10 min; 36 cycles including a denaturation step at 94 °C for 30 s, annealing at 53.2 °C for 35 s, and extension at 72 °C for 90 s; and a final extension at 72 °C for 5 min. The amplification protocol for the PsaF/R primer was performed as follows [18]: 1 cycle at 94 °C for 4 min; 36 cycles at 94 °C for 30 s, 55 °C for 30 s, and 72 °C for 30 s; and a final extension cycle at 72 °C for 10 min. A sterile water control without DNA was included in each PCR batch. In total, 5 μL of PCR products was electrophoresed on 1% TAE agarose gels for 30 min and observed using a gel imaging analysis system with ImageLab software version 6.0 (Bio-Rad Laboratories, Inc., Hercules, CA, USA). PCR products from the final selected primers were sequenced (Sangon biotech, Shanghai, China) and aligned with the known sequences in the National Center of Biotechnology Information (NCBI; https://www.ncbi.nlm.nih.gov, accessed on 6 November 2024). Nucleotide sequences and homology analysis were displayed using the SnapGene Viewer 8.0 software. Furthermore, the amplification reaction conditions of ten Psa strains and ten other *Pseudomonas* strains for specificity were consistent with those described above, and sterile ddH_2_O served as a negative control.

### 2.4. Selection of Propidium Monoazide Treatment Conditions

PMA was dissolved in 1 mL of dimethylsulfoxide (DMSO) to create a PMA mother solution with a concentration of 1 mg/mL (2 mM) before being stored at −20 °C away from direct light. For the assays, 500 µL of live and heat-treated Psa MJ2107 suspensions was supplemented with PMA to obtain a final dye concentration of 5, 10, 15, 20, 25, 30, 35, and 40 μg/mL, respectively. After dye addition, the samples were wrapped in tin foil and gently mixed 2 or 3 times in darkness at room temperature for 0, 2, 4, 6, 8, 10, and 12 min. PMA-treated and PMA-non-treated samples were subsequently exposed to a halogen lamp (650 W) for 0, 5, 10, 15, 20, 25, and 30 min. In addition, the samples were placed on ice at a distance of 20 cm away from the lamp and were mixed gently every 5 min during exposure. After exposure, the bacterial cells were centrifuged (Thermo Fisher Scientific, Waltham, MA, USA) for 2 min at 13,000× *g*, cleaned twice with sterile ddH_2_O, and then suspended with 500 μL of sterile ddH_2_O to extract total DNA as a template for further DNA extraction and amplification. The samples treated or non-treated with PMA were amplified using PCR with the PsaF/R primers as described in Section 2.3.

### 2.5. False Positive Verification of the Detection Assay Using Propidium Monoazide

The prepared bacterial suspensions with concentrations of 10^7^, 10^6^, 10^5^, 10^4^, 10^3^, 10^2^, and 10^1^ cfu/mL were used as templates to detect the heat-lethal bacteria using traditional PCR, PMA–PCR, and the spread plate method to verify the false positives using PMA–PCR.

### 2.6. Detection of Different Ratios of Live/Dead Bacteria Mixture Using Propidium Monoazide

Suspensions of dead and live bacteria, each with a concentration of 1 × 10^7^ cfu/mL, were prepared as described in Section 2.1. Various proportions of live and dead bacteria were then mixed to achieve the following ratios of live bacteria in 1 mL of bacterial suspension: 0%, 0.1%, 1%, 10%, 50%, and 100% (live/dead bacteria were mixed as 1:1, 1:9, 1:99, 1:999, and 0:1). The detection was conducted using the established PMA–PCR method.

### 2.7. Actual Sample Detection

Branch samples showing suspected symptoms of kiwifruit canker disease were collected from 10 different kiwifruit planting sites (Table 2). The samples of asymptomatic leaves or branches under rain-shelter cultivation and open-air cultivation for seasonal detection were collected from four orchards in Dujiangyan City, Sichuan using the random sampling method (in total, there were 1040 samples). The leaves of kiwifruit began to drop due to brown spot disease in September, and no samples were collected from this time. In late November, pruning and binding on the kiwifruit branches were carried out in the field. To minimize damage as much as possible, sampling was avoided to reduce sample collections since this fieldwork could cause wounds to the trees and potentially induce further diseases. Branches were taken at a length of 15 cm from the lower end of the morphology. After these samples were surface washed with running water and 75% alcohol, they were cut into small pieces (5 × 5 mm), treated with 75% alcohol for 30 s and 10% sodium hypochlorite for 2 min, and then washed with sterile water thrice. Three grams of treated small fragments was placed in 50 mL tubes, 30 mL of sterile PBS was added, and then, they were shaken in a 150 rpm/min shaker for 3 h at 25 °C. The extraction was centrifuged for 10 min, and the supernatant was poured away before being precipitated with 1mL sterile water thrice for the traditional PCR, PMA–PCR, or spread plate method. Three replicates were set for each site.

## 3. Results

### 3.1. Thermal Lethal Time Identification of Psa Cells

Heat treatments can result in dead bacteria with various degrees of cell membrane permeability. To confirm the complete inactivation of Psa strain MJ2107 using the PMA–PCR assay for specifically detecting live bacteria, the spread plate method was used (Table 3). The results showed that the number of bacterial colonies decreased with the increase in treatment time. MJ2107 exposed to a temperature of 100 °C for 15 min or more showed no cellular growth in LB agar medium, confirming bacterial cell inactivation and ensuring complete non-viability in the samples.

### 3.2. Comparison of the Amplification and Sensitivity of Specific Primers

**Figure 1:**
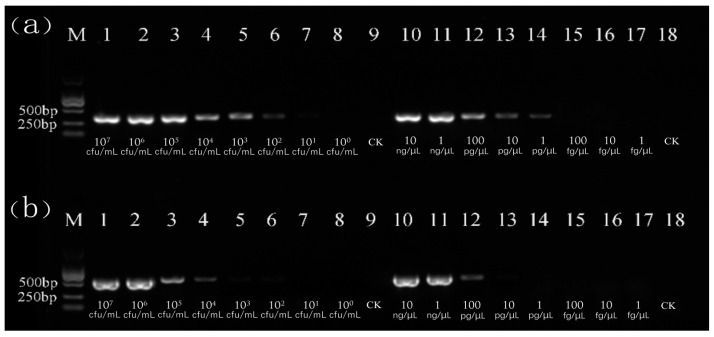
Comparison of the amplification and sensitivity of the effector *hopZ3* gene (**a**) and effector *hopZ5* gene (**b**) specific primers of Psa. Lanes 1–8: bacterial suspension concentrations. Lanes 10–17: DNA concentrations. Lanes 9 and 18: a negative control of sterile water (CK). M: DL2000 marker.

**Figure 2:**
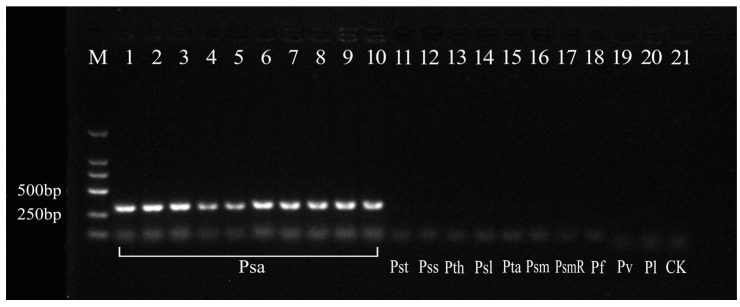
Specificity detection of the PsaF/R primer. Lanes 1–10: Psa strains. Lanes 11–20: Pst for *P. s.* pv. *tomato*, Pss for *P. s.* pv. *syringae*, Pth for *P. s.* pv. *theae*, Psl for *P. s.* pv. *lachrymans*, Pta for *P. s.* pv. *tabaci*, Psm for *P. s.* pv. *mori*, PsmR for *P. s.* pv. *morsprunorum*, Pf for *P. fluorescens*, Pv for *P. viridiflava*, and Pl for *P. lurida*. Lane 21: a negative control of sterile water (CK). M: DL2000 marker.

**Figure A1:**
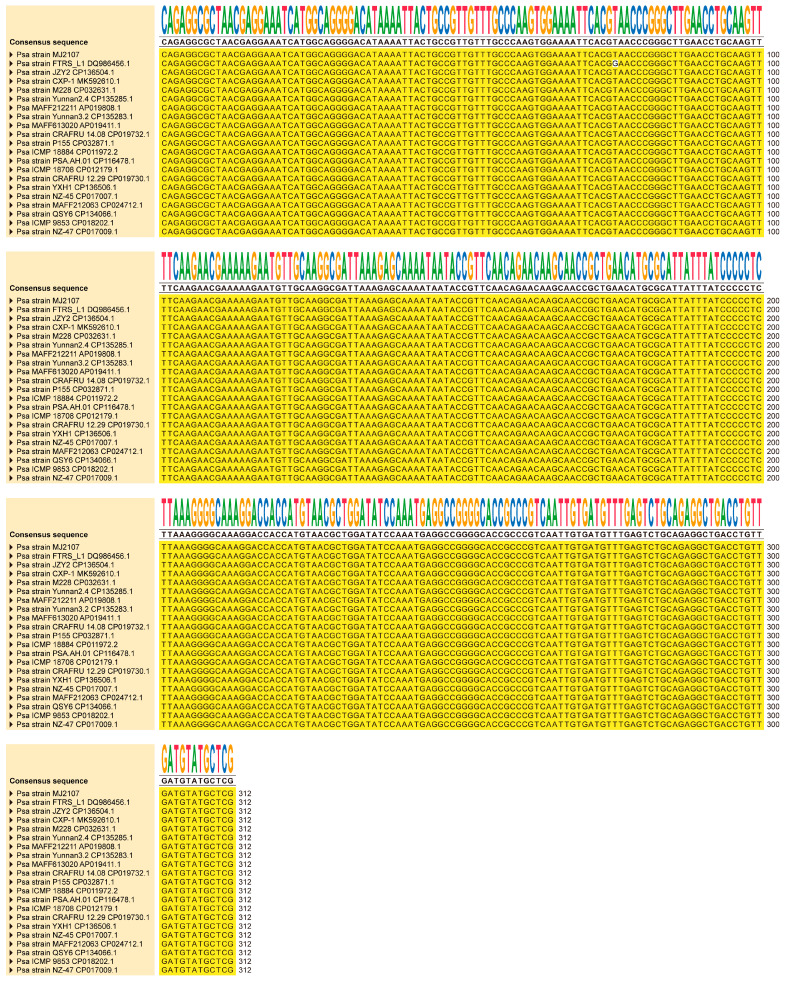
Nucleotide sequences and alignments of the partial *hopZ3* gene according to the PsaF/R primer.

Bacterial suspensions and DNA exactions were used as templates; PsaF/R and HopZ5F/R were the specific primers, whose fragment sizes were 312 bp and 520 bp, respectively. The results are shown in Figure 1, from which the different sensitivities of PsaF/R and HopZ5F/R were observed; PsaF/R had a higher sensitivity. The lower detection limit for the bacterial suspension was determined to be 10 cfu/mL, while a detection limit for DNA was established at 1 pg/µL. Consequently, the PsaF/R primer (designed from hopZ3) was selected as the subsequent detection primer. In addition, the hopZ3 gene sequences of twenty known Psa strains were downloaded from the NCBI database along with the sequence of PCR products with a high homology, which reached 99.68–100% (Table 4 and Figure A1). Twenty strains were amplified for specificity detection using the PsaF/R primer. The results showed that all Psa strains could amplify a specific band of 312 bp, while the other 10 non-target strains did not produce any amplified bands (Figure 2). It showed that the PsaF/R primer had a good specificity and high sensitivity.

### 3.3. Determination of Experimental Conditions for Propidium Monoazide in Viability Assays

**Figure 3:**
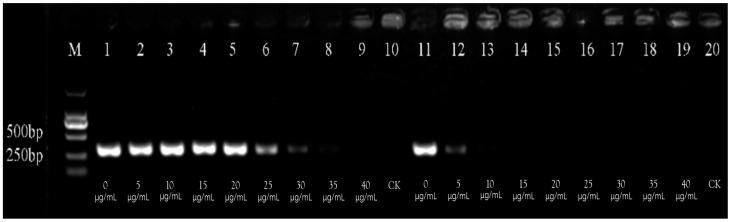
Screening of PMA concentration for detecting Psa. Lanes 1–9: the PMA concentrations of detecting viable bacterial cells. Lanes 11–19: the PMA concentrations of detecting dead bacterial cells. Lanes 10 and 20: a negative control of sterile water (CK). M: DL2000 marker.

The concentration of PMA used in this study was determined by detecting Psa at a concentration of 10^7^ cfu/mL (Figure 3). The findings indicated that, as the concentration of PMA increased, more PMA bound to the DNA of heat-killed bacterial cells, leading to a gradual weakening and eventual disappearance of the brightness of the target fragment. Specifically, when the concentration of PMA exceeded 10 μg/mL, the brightness of the target fragment began to diminish significantly, suggesting that PCR amplification for heat-lethal bacterial cells was completely inhibited. Consequently, it was established that the maximum PMA concentration required for the complete inhibition of PCR amplification for heat-lethal bacterial cells is 10 μg/mL.

However, it should be noted that excessively high PMA concentrations could adversely affect viable bacteria, and its effect would be increasingly pronounced with rising concentrations of PMA. When the concentration remained at or below 20 μg/mL, there was no significant impact on live bacteria, as evidenced by the consistent brightness levels in the target fragments, indicating that PMA did not inhibit DNA amplification from live bacteria. In contrast, once PMA concentrations surpassed 20 μg/mL, its effects on viable bacteria became evident, and their expansion was notably inhibited. Therefore, the optimal concentration for PMA–PCR to detect viable Psa cells was established at 10 μg/mL.

**Figure 4:**
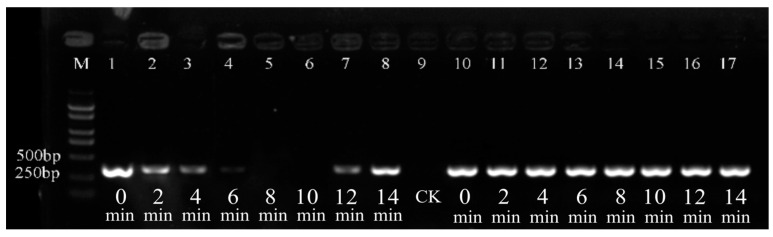
Effect of different PMA incubation times on Psa. Lanes 1–8: the dark incubation time of heat-lethal bacteria. Lanes 10–17: the dark incubation time of live bacteria. Lane 9: a negative control of sterile water (CK). M: DL2000 marker.

The dark incubation time of the bacterial suspension was evaluated (Figure 4). The findings indicated that the brightness of the target fragments remained constant throughout all dark incubation treatments. In contrast, the brightness of the target fragment of dead bacterial cells exhibited variability in relation to the duration of dark incubation. Specifically, when the dark incubation time was less than 8 min, the brightness of the target fragment diminished. At dark incubation times of 8 and 10 min, the target fragment completely disappeared. However, when the dark incubation time exceeded 10 min, the target fragment reappeared. Therefore, when PMA was used to treat the bacteria in darkness for less than 8 min or for more than 10 min, it failed to completely suppress the DNA of the dead bacteria. However, a dark incubation period of 8 to 10 min could completely inhibit the amplification of the DNA of dead bacteria.

**Figure 5:**
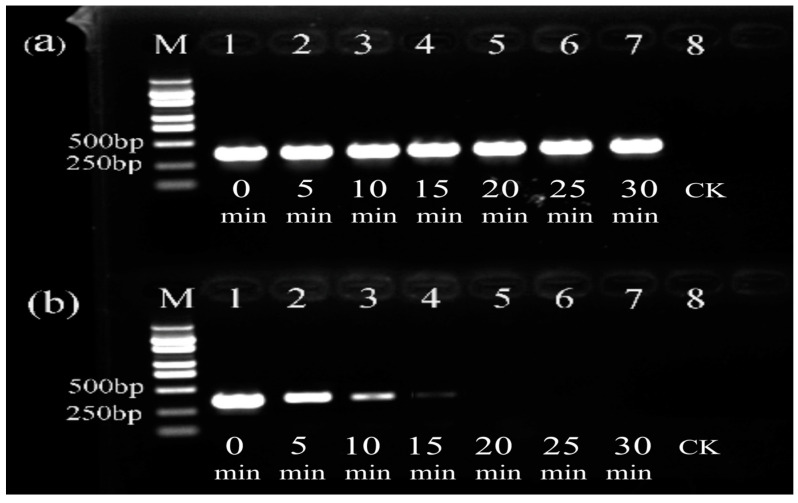
The effect of different PMA exposure times on Psa causing kiwifruit canker disease. (**a**) Viable bacteria; (**b**) heat-killed bacteria. Lanes 1–7: exposure time. Lane 8: a negative control of sterile water (CK). M: DL5000 marker.

The bacterial suspension with PMA was exposed for different times (Figure 5). The brightness of the target fragment of the live bacteria did not change over time (Figure 5a). In contrast, as the exposure time increased, the brightness of the target fragment of heat-killed bacteria decreased gradually (Figure 5b). At 20 min or longer, the target fragment completely disappeared. The results indicated that the optimal exposure time for the PMA–PCR detection of heat-killed Psa bacterial cells was 20 min, which fully suppressed the PCR amplification of dead bacteria while having no impact on live ones.

### 3.4. Detection of the Live Bacterial Cells in the Mixed Samples with Dead Cells Using Propidium Monoazide

**Figure 6:**
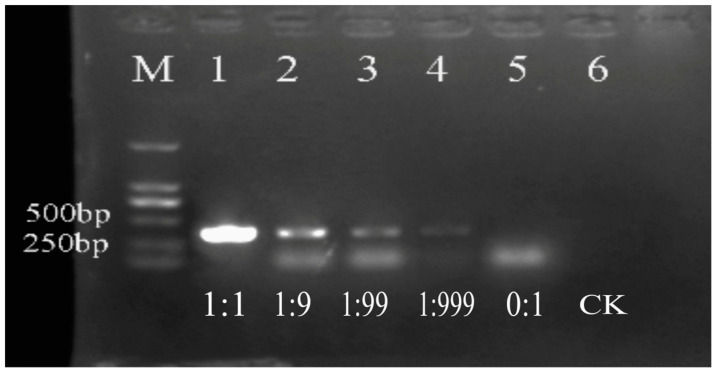
PMA–PCR detection of different proportions of dead and live bacteria in a mixed system. Lanes 1–5: the proportions of the live bacteria and the heat-lethal bacteria. Lane 6: a negative control of sterile water (CK). M: DL2000 marker.

In mixed samples of heat-lethal and live bacterial cells with different proportions, the detection results of the live bacteria can be seen in Figure 6. We found that live bacterial cells in the mixed samples with different proportions of dead cells were all positive. As the proportion of dead bacteria increased, the target fragments darken. The target disappeared when the percentage of dead bacterial cells accounted for 100%. It showed that the PMA–PCR assay could be used to detect the viable bacteria in a mixed cell suspension with dead bacteria.

### 3.5. False Positive Verification of the Assay Using Propidium Monoazide

Inactivated Psa cells were detected using the traditional PCR, PMA–PCR, and spread plate methods to verify whether false positives could be generated by PMA–PCR (Table 5). The detection results of PMA–PCR and spread plate counting were negative, while the conventional PCR showed a positive result. This indicated that PMA–PCR could distinguish between dead and viable Psa cells; the reliability of the results was verified using the spread plate method.

### 3.6. Detection of Actual Samples in the Field Using Propidium Monoazide

Ten branch samples exhibiting pronounced canker symptoms were collected from the field, and viable Psa cells were detected using PMA–PCR (Table 6). The results indicated that the control treatments of PMA–PCR were negative, demonstrating that the DNA amplification of dead Psa cells in the heat-killed samples was completely inhibited and could not be detected. Samples obtained from Chongzhou (B8) and Aba (B9) yielded negative results for both the PMA–PCR and spread plate culture methods. Conversely, the remaining eight samples were positive according to the PMA–PCR and spread plate culture methods. All samples were positive except for those from Chongzhou according to normal PCR. These findings suggested that the samples from Chongzhou were uninfected with Psa, while those from Aba contained dead Psa cells, whose nucleic acids were still detectable. Consequently, PMA–PCR served as a rapid and precise method for detecting live pathogen cells in kiwifruit branch samples, offering an effective alternative to traditional spread plate culture methods.

Additionally, the viable Psa populations on the branches and leaves of kiwifruit were detected using the PMA–PCR method for both rain-shelter and open-air cultivation (Table 7). The results indicated that the detection rates of live bacteria on branches and leaves varied across different periods as well as between the two cultivation modes. From January to April, the detection rate of live bacteria in branches under open-air cultivation was significantly higher than that observed under rain-shelter conditions, with a peak occurring in April (which was also noted in leaves). During May to December, there were minimal differences in the detection rates of viable Psa bacteria between the two cultivation methods. At the same time, it was noteworthy that, from January to April, particularly under rain-shelter conditions, the detection rate remained higher. There was a period during May in which the viable Psa bacterial cells declined, and the prevalence of kiwifruit canker disease started to diminish. These findings suggest that the PMA–PCR method effectively monitors dynamic changes in Psa throughout this disease cycle while filtering out influences from dead bacterial cells.

## 4. Discussion

*Pseudomonas syringae* pv. *actinidiae* is the pathogen causing bacterial canker disease in red-fleshed and yellow-fleshed kiwifruit. This pathogen has inflicted significant economic damage in countries, including Italy and New Zealand, where kiwifruit constitutes a primary agricultural product, as well as causing substantial losses in France, Spain, Portugal, Chile, China, South Korea, and Japan. In a short time, bacterial canker has emerged as a widespread plant disease, causing great losses to kiwifruit production systems [12,18,40,41,42,43]. The effective diagnosis and detection of pathogens are crucial steps in controlling bacterial diseases. As a phytosanitary pathogen, the accurate detection of live *Pseudomonas syringae* pv. *actinidiae* to prevent its dissemination is particularly important. Furthermore, the importance of early detection and prevention strategies concerning kiwifruit canker disease is supported [44,45,46]. Fround et al. [47] found that the timely improvement in management measures after the first detection of Psa in kiwifruit orchards could help improve productivity by establishing a multivariate linear regression interpretation model. Cacioppo et al. [48] measured soil and climate parameters using two sensing systems (’OsiriS’ and ’OttaviO’) to predict Psa infection for copper treatment time and found that this early diagnosis resulted in a 60% reduction in canker symptoms and reduced treatment costs [49]. In previous studies, multiple assays, containing the spread plate count method and molecular biology, were widely used to detect the pathogen of kiwifruit canker disease. Among these molecular biology methods, in 1994, Scortichini [50] identified *Pseudomonas syringae* pv. *actinidiae* based on the physiological morphological characteristics and pathogenicity. Subsequently, diversified techniques such as PCR restriction fragment length polymorphism analysis, real-time quantitative PCR, and nested PCR were used to identify and detect Psa [17,18,23,42,51,52,53,54]. However, there are few reports on the detection of live bacteria, and the results are often susceptible to false positives. The accurate and sensitive identification of pathogenic bacterial conditions is crucial for developing and implementing effective control measures against plant diseases. In particular, the absence of false positive results is paramount when testing quarantine agents because samples showing false positives are considered to pose no threat to plant disease epidemiology. Although false positive samples themselves are not a threat to the plant, the occurrence of false positive samples may mislead kiwifruit growers when judging the prevalence of the disease, leading to the adoption of inappropriate prevention and control strategies, such as the overuse of agricultural chemicals, as well as unreasonable pruning and quarantine. These unnecessary strategies may cause growers to suffer significant economic losses in terms of control cost, yield loss, and market sales as well as have a significant negative impact on the economic benefits of the kiwifruit industry, which is not conducive to its sustainable development [25,55,56]. Furthermore, epidemiological studies of plant diseases necessitate more reliable data regarding pathogenic bacteria populations to effectively model their occurrence and spread.

Propidium monoazide (PMA) is a fluorescent dye that is capable of inhibiting the DNA amplification of dead bacterial cells during PCR processes. The underlying principle involves enhanced membrane permeability in dead bacterial cells, and PMA can penetrate these membranes and bind to the DNA of dead cells, thereby preventing its amplification during PCR [57]. Combining PMA and polymerase chain reaction (PCR) for pathogen detection effectively avoids the false positives that are commonly associated with normal PCR, accurately allowing for the diagnosis of plant diseases and facilitating the formulation of effective management strategies. Consequently, leveraging the characteristics of PMA, a rapid and efficient detection method for Psa, via PMA–PCR can be established.

PMA–PCR has been extensively utilized in detecting foodborne pathogenic microorganisms as well as medical pathogens [58,59,60,61,62]. It has also been reported that PMA–PCR is applied to detect plant pathogenic bacteria. Gao et al. [63] and Yu et al. [30] established a method for detecting *Ralstonia solanacearum*, which includes viable but nonculturable (VBNC) bacteria, using PMA combined with PCR (PMA–PCR). Zhao et al. [64] demonstrated that live cells responsible for bacterial fruit spot in watermelon were detectable via PMA–PCR. Additionally, Yu et al. [35] developed a PMA–PCR method specifically designed to assess cell viability in *Streptosporium oryzae* associated with rice bacterial stripe disease, effectively distinguishing between live and dead cells of *Streptosporium oryzae*.

Among the molecular biology techniques developed for Psa, Zhou et al. [65] developed a method combining ethidium monoazide (EMA) with quantitative PCR (qPCR) to detect live Psa. Although EMA and PMA exhibited similar functionalities, a small amount of EMA molecules could permeate the cell membrane of viable bacteria and were toxic to the cells, potentially compromising the reliability of the results. In our study, we employed PMA to effectively eliminate the errors associated with EMA. Xiao et al. [66] utilized a PMA–qPCR methodology (with mRNA as template) to detect live Psa-V, which was an important pathogen responsible for kiwifruit canker disease in Shaanxi Province, with a higher PMA concentration at 105 μg/mL compared to the concentration of 10 μg/mL used in our study. This discrepancy may stem from variations in template treatment used during detection processes. Moreover, when the concentration of pathogens and other bacterial compositions in actual samples from the field are unknown, PMA is unreliable for the quantitative determination of cell viability [67]. Nevertheless, PMA is reliable for qualitative determination in dead vs. live cells, and our findings aligned closely with those observed in other Gram-negative bacteria using PMA–PCR. Of all the tested strains in this study, Pss and Pv were reported to cause diseases with symptoms that were similar to canker symptoms in kiwifruit by Psa, although the incidence rates were relatively low [17,28,68,69,70]. For disease treatments, there are some differences between them and Psa. The violent reactions are applied for controlling Psa, while moderate management is applied for Pss and Pv because of their different harmful degrees and infrequent distribution in kiwifruit orchards [28,71]. The rest of the tested strains are not pathogenic bacteria in kiwifruit, although they are closely related to or co-colonized with Psa. Therefore, the correct distinction between Psa and other similar bacteria is particularly important. In our study, the specificity of the PsaF/R primer was very robust, effectively distinguishing Psa from other pathogenic bacteria of *Pseudomonas (syringae*) populations (Figure 2). These findings are largely in agreement with previous studies [18,28]. Furthermore, with effector *HopZ3* as the specific target, a lower limit of detection for bacterial suspensions was observed at 10 cfu/mL with DNA concentrations at 1 pg/μL, which enabled effective identification even at low concentrations of living cells, which is suitable for samples exhibiting low infection levels.

To further assess practical applicability, ten field samples suspected to have kiwifruit canker disease were selected for detection. Eight out of the ten suspected samples in the field showed positive results based on PMA–PCR, which was more specific than normal PCR and could avoid false positives. Simultaneously, the consistency and dependability of the PMA–PCR detections were confirmed using the spread plate method. The PMA–PCR assay also offered a shorter detection time than the spread plate counting method and could reliably indicate whether the suspected sample contains live pathogenic bacteria. Furthermore, the detection of viable Psa on asymptomatic kiwifruit tissues using PMA–PCR was developed in our study. Due to low temperature and high humidity as well as rainy weather and freezing damage, the resistance of the tree is decreased, which is conducive to the invasion and rapid propagation of Psa [72,73].

During January to April, the detection rates of viable Psa were at a high level due to the amount of Psa that had invaded the kiwifuit [74,75,76]; a significant decrease occurred in May because of the rising temperatures under open-air cultivation [77], while there was only a slight difference under rain-shelter cultivation in this study. Although there was a little recovery in autumn [72], it still remained at a low level. All the phenomena were substantially consistent with the occurrence pattern of kiwifruit canker disease [73], and it was proven that the PMA–PCR method could accurately determine the occurrence of field diseases in real time. Therefore, PMA–PCR is a technique that can be used to detect *Pseudomonas syringae* pv. *actinidiae* with high specificity, efficiently monitor the early occurrence and development of kiwifruit canker disease, and prevent long-distance transmission through pollens, fruits, seedlings, and other ways.

## 5. Conclusions

In this study, the specificity and sensitivity of the PsaF/R primer were detected, which could specifically detect Psa. In addition, we successfully established PMA–PCR as a method for detecting live Psa cells, and actual field samples could be detected to prove the feasibility of this method. This approach not only offers a rapid and targeted means of identifying live Psa cells but also serves as a reference for the swift preliminary screening of this pathogen’s activity in laboratory settings. Furthermore, it provides a theoretical foundation for the prevention and control of kiwifruit canker disease and has good application prospects.

## Figures and Tables

**Table 1:** The primer sequences for the molecular identification of *Pseudomonas syringae* pv. *actinidiae*.

Primer Name	Gene	Sequence (5′→3′)
PsaF	*hopZ3*	CAGAGGCGCTAACGAGGAAA
PsaR		CGAGCATACATCAACAGGTCA
HopZ5F	*hopZ5*	TCACTCCTAGACTGGAATAC
HopZ5R		GGCTATCATGAAGGCTGTCA

**Table 2:** Actual sample source information.

Scheme	Site	Latitude (°N)	Longitude (°E)	Host or Cultivar
B1	Xichang	28.09	102.04	Donghong
B2	Qionglai	30.52	103.717	Hongyang
B3	Pujiang	30.26	103.62	Hongyang
B4	Yaan	30.15	102.96	Hongyang
B5	Dujiangyan	30.92	103.61	Hongyang
B6	Dujiangyan	30.92	103.61	Hongyang
B7	Dujiangyan	30.92	103.61	Hongyang
B8	Chongzhou	31.55	106.17	Hongyang
B9	Aba prefecture	30.55	103.64	Hongyang
B10	Guangyuan	30.93	103.42	Cuiyu

**Table 3:** Screening of the inactivation time of viable bacteria.

Heated Time (min)	Number of Bacterial Colonies (cfu/mL)
* CK (0)	1 × 10^7^
11	10
12	7
13	3
14	2
15	0
16	0
17	0
18	0
19	0
20	0

* CK represents that the Psa strain MJ2107 is not heated at 100 °C.

**Table 4:** The percent homology analysis between Psa strain MJ2107 and the other twenty Psa strains at the PsaF/R primer binding sites.

Strain	Genbank ID	Percent Homology (%)
FTRS_L1	DQ986456.1	99.68
JZY2	CP136504.1	100
CXP-1	MK592610.1	100
M228	CP032631.1	100
Yunnan2.4	CP135285.1	100
MAFF212211	AP019808.1	100
Yunnan3.2	CP135283.1	100
MAFF613020	AP019411.1	100
CRAFRU14.08	CP019732.1	100
P155	CP032871.1	100
ICMP18884	CP011972.2	100
PSA.AH.01	CP116478.1	100
ICMP 18708	CP012179.1	100
CRAFRU12.29	CP019730.1	100
YXH1	CP136506.1	100
NZ-45	CP017007.1	100
MAFF212063	CP024712.1	100
QSY6	CP134066.1	100
ICMP 9853	CP018202.1	100
NZ-47	CP017009.1	100

**Table 5:** Comparison of normal PCR, PMA–PCR, and spread plate counting methods for the detection of dead Psa bacteria.

Psa Concentration /cfu·mL^−1^	Normal Polymerase Chain Reaction	PMA–Polymerase Chain Reaction	Spread Plate Counting /cfu
10^7^	+	-	0
10^6^	+	−	0
10^5^	+	−	0
10^4^	+	−	0
10^3^	+	−	0
10^2^	−	−	0
10^1^	−	−	0

Note: “+” means Psa can be detected, which is positive; “−” means Psa cannot be detected, which is negative.

**Table 6:** Detection of kiwifruit branch samples with kiwifruit canker disease in the field.

Sample	Normal PCR	PMA–PCR	Plate Culture	PMA–PCR (CK)
B1	+	+	+	−
B2	+	+	+	−
B3	+	+	+	−
B4	+	+	+	−
B5	+	+	+	−
B6	+	+	+	−
B7	+	+	+	−
B8	−	−	−	−
B9	+	−	−	−
B10	+	+	+	−

Note: “CK” represents heat-killed Psa cells. “+” means Psa can be detected, which is positive; “−” means Psa cannot be detected, which is negative.

**Table 7:** Detection of viable *Pseudomonas syringae* pv. *actinidiae* in kiwifruit branch samples over one year in the field using PMA–PCR.

Month/Time	Rain-Shelter Cultivation		Open-Air Cultivation	
	Branch	Leaf	Branch	Leaf
January	4/32	-	12/32	-
February	5/32	-	14/32	-
March	4/32	1/32	14/32	5/32
April	6/32	13/32	17/32	22/32
May	3/32	2/32	2/32	3/32
June	4/32	1/32	2/32	3/32
July	1/12	0/12	1/12	0/12
August	2/24	0/12	2/24	0/12
September	4/40	-	2/40	-
October	5/40	-	4/40	-
November	3/40	-	6/40	-
December	2/20	-	3/20	-
Total	43/368	17/152	77/368	33/152

Note: “-” means no samples because of seasonal leaf falling.

## Data Availability

Data are available upon request from the authors.

## Appendix A

**Table A1:** Psa strains used in this study.

Number	Strain	Host or Cultivar	Tissue	Site
1	MJ2107	Hort16A	leaves	104.13° E, 31.39° N
2	CX1101	Hongyang	branches	105.93° E, 31.73° N
3	AB2101	Xuxiang	leaves	103.43° E, 30.94° N
4	DH2101	Hongyang	leaves	103.70° E, 31.00° N
5	DS2102	Hongyang	leaves	103.73° E, 31.02° N
6	DF2101	Hongyang	leaves	103.74° E, 31.02° N
7	MP2101	Hongyang	leaves	104.13° E, 31.39° N
8	MN2101	Hongyang	canes	104.13° E, 31.39° N
9	QL0102	Hongyang	branches	103.57° E, 30.38° N
10	YA1801	Hongyang	branches	102.94° E, 30.16° N
